# Determining space requirements for small and sick newborns and their mothers in health facilities: a systematic review

**DOI:** 10.7189/jogh.15.04313

**Published:** 2025-10-17

**Authors:** Natalie Strobel, Georgia Whisson, Derek Swe, Rajesh Mehta, Amy Budrikis, Karen Edmond

**Affiliations:** 1 Kurongkurl Katitjin, Edith Cowan University, Perth, Australia; 2 Public Health Foundation of India and Indian Institutes of Public Health, New Delhi, India; 3 World Health Organization, Geneva, Switzerland

## Abstract

**Background:**

There are currently no World Health Organization and United Nations Children Fund benchmarks or ‘norms’ for scaling up small and sick newborn (SSN) service delivery in health facilities in low- and middle-income countries (LMICs). Specifically, there is limited evidence on optimal bed space requirements in SSN units such as special care nurseries and neonatal intensive care units (NICUs). Through this systematic review, we aimed to gather evidence on the optimal space requirements needed for SSNs and their mothers in health facilities, particularly at level 2 district hospitals.

**Methods:**

We included simulation, experimental, and observational studies, as well as guidelines and formal expert opinion processes that described the optimal bed space for SSN units within any health facility. We searched Medline (*via* Ovid), EMBASE (*via* Ovid), CENTRAL (*via* the Cochrane Library), CINAHL (*via* EBSCO), and LILACS from inception to October 2023. We assessed the quality of included studies using the RoB 2, ROBINS I, ROBINS E, and AGREE-II tools. We narratively described and synthesised all data.

**Results:**

We identified 10 574 studies and, after deduplication, screened 8453 titles and abstracts and assessed, followed by the full texts of 125 studies for eligibility. We included 12 reports which discussed three guidelines and one simulation-based study. All four came from high-income countries. Only one guideline specified the required bed space and floor space to be 2.8 m^2^ and 14 m^2^ per bed in an open bay unit, respectively. One study defined the mother-NICU bed space requirements for a mother and her infant as 20 m^2^ (inclusive of the bathroom), while another stated it to be 28 m^2^ (exclusive of the bathroom).

**Conclusions:**

We found no studies describing space requirements for SSN units in LMICs and limited evidence for the optimal space requirements for open bay and mother-NICU beds in high-income countries. Much of the data reported was too heterogeneous to combine. More research, especially simulation studies, is needed to establish optimal space requirements for SSN units, including benchmarks that demonstrate functionality and the impact on infection control.

**Registration:**

PROSPERO: CRD42022378329.

Small and sick newborns (SSNs) are neonates who are born premature (<37 weeks), low birthweight (<2.5 kg), small for gestational age, or have neurological conditions (*e.g.* birth asphyxia, hypoxic ischaemic encephalopathy, neonatal seizures), infections (*e.g.* meningitis or sepsis), or nutritional problems (*e.g.* growth faltering and failure or feeding difficulties) [1,2]. Their survival, health, and development are areas of concern for many countries; however, limited resources and infrastructure often hamper the support that they can provide to these infants.[3,4] The global Every Newborn Action Plan defines three levels of newborn care: primary care clinics (level 1), district hospitals or equivalent (level 2), and tertiary or specialist hospitals (level 3) [1,2]. The fourth global Every Newborn Action Plan target is to have at least one level 2 SSN care unit in every district (or equivalent) in every country [1]. Unfortunately, due to variations in population size, geography, birth rates, and admission policies across districts and countries, this target has been challenging to apply.

The World Health Organization (WHO) and the United Nations Children Fund have recently been developing normative data or ‘benchmarks’ for SSN service delivery in level 2 facilities in low- and middle-income countries (LMICs). This included establishing the number of beds per population of live births, time to travel to level 2 facilities, human resources, and the space required for SSN care units. In a comprehensive consultation among experts in SSN care, approximately 500 participants from 43 countries identified a model of SSN care that includes ten core components [5], including infrastructure, design, and space provision for health facilities. Understanding the optimal space requirements in level 2 special care nurseries and neonatal intensive care units (NICUs), also known as SSN units, was considered a high priority.

Two systematic reviews have investigated optimal space requirements for SSN units over the last six years [6,7]. O’Callaghan and colleagues aimed to determine NICU design features that would improve neonatal, parental, and staff outcomes [6]. As part of this review, the evidence for recommending space for individual infants, multi-cot, and private rooms was determined from three studies dating from 2004–13 [8–10], which does not include any recent evidence. There was also no attempt to pool estimates of space values. A more recent review by Zana-Taieb and colleagues investigated the minimum area requirements for a single infant's hospital room and recommended a minimum area of 18.5 m^2^ for open-bay units and 24 m^2^ for private family rooms [7]. Neither of these reviews found evidence for mother-NICUs (M-NICUs), attempted to pool estimates for space, nor were they aimed at determining the optimal space for SSN units. For this review, we considered the concept of optimal space as being the ideal space to maximise infant and family outcomes whilst also supporting staff efficiency, and from a health systems perspective, being cost-effective and adaptable. As such, we are considered ‘optimal’ as ‘what it should be’ rather than what the current space is. Thus, we conducted a systematic review to determine the optimal space requirements for SSNs and their mothers in health facilities, particularly at level 2 district hospitals.

## METHODS

We registered this review in PROSPERO (CRD42022378329) and followed the PRISMA guidelines in reporting our findings (Appendices S1 and S2 in the **Online Supplementary Document**) [11]. We employed standard methods in performing the review, as outlined in the Cochrane Handbook for Systematic Reviews [12].

### Study design

We searched for simulation studies, experimental research (*e.g.* randomised controlled trials, quasi-randomised controlled trials, and experimental designs), observational studies (*e.g.* cohort, cross-sectional, and case-control studies), guidelines, and formal expert opinion processes (*e.g.* Delphi processes or steering groups). We excluded all other study designs, abstracts, and editorial/opinion pieces, as we aimed to report on bed and floor space in SSN units, with an assumption that they would not appropriately address our phenomenon of interest or outcomes.

### Phenomenon of interest

We investigated bed space requirements for SSN units, which refers to any level of SSN care (**Table 1**) in any facility design. Facility designs could include:

**Table 1:** Levels of neonatal care*

	NICU Level	Care standards	Health facility
**Well newborn nursery**	Level 1 (I)	Provide neonatal resuscitation at delivery, postnatal evaluation and care for stable term and late premature (35–37 weeks GA) infants, and postnatal stabilisation of ill infants and moderately to extremely premature infants (<35 weeks GA) for transfer to level 2	Small hospital/health centre (level 1)
**Small and sick newborn care units/care**			
Special care nursery	Level 2 (II)	Level 1 plus provide postnatal evaluation and care for very premature (≥32 weeks gestation) and/or low birth weight (≥1500 g) infants, where moderate illness is expected to resolve rapidly and is unlikely to require urgent subspecialty care, convalescent, step-down care of infants following intensive care (Level III), brief mechanical ventilation (<24 h) or continuous positive airway pressure, and postnatal stabilisation of extremely premature (<32 weeks GA) and/or low birth weight (<1500 g) for transfer to level 3	District/provincial hospital (level 2)
NICU	Level 3 (III)	Level 2 plus provide sustained life support, comprehensive care of extremely premature (<32 weeks GA) and/or low birth weight (<1500 g) and critically ill infants of any GA and/or birth weight, access to a range of paediatric subspecialties, full range of respiratory support, including ventilation and inhaled nitric oxide, and advanced imaging capabilities with rapid interpretation available	Tertiary/referral hospital (level 3)
Regional/ advanced NICU	Level 4 (IV)	Level 3 plus provide surgical repair of complex acquired or congenital conditions (located within a health facility that has this capability), full onsite availability of paediatric subspecialties, and transport and outreach education	Tertiary/regional or national referral hospital (level 3)

GA – gestational age, NICU – neonatal intensive care unit

*Adapted from a previously published study [13].

- Multi-cot areas, also known as multi-bedrooms or open bay units: infants are cared for in a standard room, typically separated by curtains;
- M-NICU, also known as couplet care or mother-infant dyad rooms: care is provided for both mother and infant without separation, regardless of the health status of the infant;
- Private room or single-family rooms: infants are cared for in individual rooms, which may or may not include a bathroom and sleeping space for families.

### Outcomes

Our primary outcome of interest was bed space requirements and floor space needed per bed as defined by m^2^/bed or ft^2^/bed for SSN care. Secondary outcomes collected included space for adjacent aisles in m^2^ or ft^2^ and whether studies reported on space (*i.e.* yes or no) for family, physical and storage areas, staff space, charting space, and equipment and supply storage space. We collected all data for open bay units or M-NICU in SSN units. We further collected information on bed space requirements and floor space needed per bed as defined by m^2^/bed or ft^2^/bed for private rooms or single-family rooms, as well as isolation, resuscitation, and operative delivery rooms, and specialised beds. We excluded all other areas in a NICU where space may have been reported. As this study was part of a wider benchmarking process for the WHO, we consider ‘optimal’ to be ‘what it should be’ rather than what the current NICU space in hospitals is. We collected data on the tools or measures used to determine space requirements, which we refer to as evidence of recommendations. We only included papers that provided the primary outcome.

### Search strategy and selection of studies

We searched Medline (*via* Ovid), EMBASE (*via* Ovid), CENTRAL (*via* the Cochrane Library), CINAHL (*via* EBSCO), and LILACS. We reviewed the reference lists of all included articles to ensure that we captured all reports of each study. The search strategy development was supported using Systematic Review Accelerator (Appendix S1 in the **Online Supplementary Document**) [14]. The search extended from the inception of each database until October 2023, and we did not apply any language restrictions. If a study was reported in a language other than English, we used Google Translate to translate the manuscript into English. We used the Covidence software to manage all stages of the review [15].

Three independent reviewers (GW, AB, and NS) screened, deduplicated, and assessed titles and abstracts, as well as full texts and the reference lists thereof, for inclusion. A fourth author (KE) resolved disagreements. We included the papers if they met the eligibility criteria.

### Data extraction

Two authors (GW and DS) independently extracted the data on a piloted data extraction form. A third author (NS) resolved the disagreements. The following data were extracted based on how we can adequately describe the included studies, support the construction of tables and figures, and provide a synthesis (Appendix S2 in the **Online Supplementary Document**):

- Review details: title, authors, year of publication, date of last assessment, number of included papers and participants, conflict of interest of review authors, and funding details;
- Study characteristics: geographic location, country (LMIC, high-income country (HIC)), SSN unit level, and included facilities;
- Outcomes: bed space, floor space, private room or single-family room, between and/or around beds (*i.e.* aisles), other related care spaces requirements as defined by m^2^/bed or ft^2^/bed, definitions on bed space and floor space, and how the evidence of a recommendation was determined such as tools and approaches in deciding the final values.

### Assessment of methodological quality

To assess the methodological quality of the potentially included studies, we used the Risk of Bias 2 (RoB 2) tool for randomised controlled trials, Risk Of Bias In Non-randomised Studies – of Interventions (ROBINS I) tool for non-randomised studies of interventions, Risk Of Bias In Non-randomized Studies – of Exposure (ROBINS E) for exposures, and Appraisal of Guidelines for Research and Evaluation II (AGREE II) for guidelines [16–19]. Two authors (GW and DS) independently completed this process, with a third author (NS) resolving discrepancies.

### Data synthesis

We described the characteristics of space requirements in SSN units narratively, including consistency across included studies and how the evidence for a recommendation was determined. If information was not recorded as either m^2^ or ft^2^, we converted the alternative units and reported both units. Depending on whether the data could be pooled for a summary, we provided the median (interquartile range and range) for each outcome; otherwise, we described the data narratively. If the evidence were available, we described differences between NICUs with and without continuous positive airway pressure facilities, different health facility types, LMICs compared to HICs, and with and without mothers staying in the SSN unit (*e.g.* multi-cot areas *vs.* M-NICU).

## RESULTS

In total, we retrieved 10 574 studies from electronic database searches and reference screening. After removing duplicates, we screened 8453 titles and abstracts. After this stage, we assessed 125 studies for eligibility and excluded 113 (Appendices S3 and S4 in the **Online Supplementary Document**). We found no citations for ongoing papers. Overall, we included 12 studies: 11 guidelines (*i.e.* three guidelines and their serial updates) [10,20–29] and one simulation study [30] (**Table 2**). Of the 11 guidelines: seven from the USA [10,20–25], two from Spain [26,27], and two from Chile [28,29] (Appendices S7–10 in the **Online Supplementary Document**). All guidelines were published in HICs. We focussed on the most recent versions of these serial updates: the current guidelines for the USA [25], Chile [29], and Spain [27]. All guidelines incorporated expert consensus, as well as a mix of published literature, experience, and suggestions to develop evidence for their recommendations (**Table 2**). However, it is unclear what evidence (*e.g.* literature and/or consensus) was used to make the final recommendations. Overall, the three guidelines offer recommendations for level 2–4 [29], level 3–4 [25], and level 1–3 SSN units [27]. However, the actual reporting of space for different SSN unit levels was not consistent (**Table 2**, **Table 3**). For the simulation study, the optimal space recommendation was determined by an expert group based on the results of functional experiments completed by 21 clinicians [30].

**Table 2:** Characteristics of included guidelines

Study, year, reference	Type of study	Aim	Country	NICU setting	Evidence of recommendation
Hignett et al., 2010 [30]	Simulation	Determine the spatial requirements for clinical activities in an individual NICU cot (incubator) space.	UK	NICU (unspecified)	Task analysis, functional space experiments, and expert opinion
Gracia et al., 2013 [27]	Guideline	Define the level of care of each health centre in our country, as well as the health and technical requirements by levels of neonatal care, and to optimise the location of resources.	Spain	NICU Levels I, II, III	Expert committee consensus
Bajaña et al., 2021 [29]	Guideline	The purpose is to update the recommendations on the organisation, characteristics, and operation of neonatology services or units, to serve as an orientation and guide for the design and management of neonatal care in public and private health care centres in the country.	Chile	NICU Level I, II, III, and IV	Neonatology advisory working group, published literature*, expert recommendations.
Altimier et al., 2023 [25]	Guideline	The purpose of this committee is to complement the above documents by providing health care professionals, architects, interior designers, state health care facility regulators, and others involved in the planning of NICUs with a comprehensive set of standards based on clinical experience and an evolving scientific database.	USA	NICU Levels III, IV	Expert committee consensus, incorporating new research findings*, experience*, and suggestions*.

NICU – neonatal intensive care unit

*It is unclear whether this evidence was used to create the recommendation for space.

**Table 3:** Space requirements in a NICU

	Bed size and/or minimum area	Adjacent aisle	Family physical space	Family storage space	Staff space	Charting space	Equipment and supply storage
**Optimal space requirements for multi-bed infant rooms**							
Gracia et al., 2013 [27]	Bed size: NR; minimum surface area (no other details); Level 1: 1.5–2 m^2^ (16–21.5 ft^2^), Level II: 4–5 m^2^ (43–54 ft^2^), Level III: 9–11 m^2^ (97–118.5 ft^2^)		Yes				
Altimier et al., 2023 [25]	Bed size intensive care: 2.8 m^2^ (30 ft^2^); Level III/IV:14 m^2^ (150 ft^2^) clear floor space†	1.2 m (4 ft) (2.4 m (8 ft) between cots)	Yes	Yes	Yes	Yes	Yes
**Optimal space requirements for couplet care rooms**							
Gracia et al., 2013 [27]			Yes				
Bajaña et al., 2021 [29]	Level III/IV: 20m^2^ (215 ft^2^)*		Yes	Yes		Yes	Yes
Altimier et al., 2023 [25]	Level III/IV: 14 m^2^ (150 ft^2^) clear floor space† for the NICU infant and 14 m^2^ (150 ft^2^) for the mother 28 m^2^ (300 ft^2^)	2.4 m (8 ft) between beds	Yes	Yes	Yes	Yes	Yes

NICU – neonatal intensive care unit, NR – not reported

*Inclusive of crib, caregiver bed, bathroom with a shower, and storage for personal belongings.

†Clear floor space is defined as the space available for functional use, including a parent to stay seated, reclining or fully recumbent and excludes other defined spaces such as plumbing fixtures, anterooms, vestibules, toilet rooms, closets, lockers, wardrobes, fixed-based cabinets, and wall-hung counters.

### Results for bed and floor space

Only the USA guidelines reported a bed space of 2.8 m^2^, which was consistent across all of the previous versions of thereof (**Table 3**; Appendix S8 in the **Online Supplementary Document**), and provided data on both multi-cot and M-NICU space sizes (**Table 3**). The described space requirements for a multi-cot area have increased from 11.2 m^2^ in 1991 to 14 m^2^ in 2020 per bed for a standard NICU. Results for bed space for M-NICUs were provided in the 2020 and 2023 guidelines, which described the space required as 14 m^2^ each for a mother and infant (28 m^2^ total) [24,25]. The space requirement described by the Chilean guidelines for an M-NICU was 20 m^2^, which included bathrooms and storage of personal belongings (health facility level unspecified) [29]. The Spanish guidelines recommend 4–5 m^2^ per bed in a multi-cot area for a special/intermediate NICU [27]. We were unable to pool and summarise information as the definitions provided in each guideline were not homogeneous.

We also collected information on whether the guidelines described space requirements for family activities, storage space, staff space, charting space, and equipment and supply storage. Only the USA guidelines [25] described requirements for the additional spaces for both multi-cot areas and M-NICUs (**Table 3**). Other optimal space requirements were defined for single-family rooms, and extra space requirements for specialised, high-acuity beds (*e.g.* isolation or resuscitation rooms) were provided in all guidelines (Appendix S9 in the **Online Supplementary Document**) [25,27,29].

The simulation study employed functional space experiments within a full-scale mock-up of a multi-cot SSN unit, incorporating equipment and furniture from a SSN unit at a London teaching hospital [30]. The parameters for an individual SSN unit bed space ranged from a minimum of 12.4 m^2^ (134 ft^2^) to a maximum of 14.85 m^2^ (160 ft^2^). Following the simulations with multiple participants (21 clinical neonatal unit staff) and expert consultation, 13.5 m^2^ per bed was considered the optimal space recommendation [30].

### Assessment of methodological quality

As we retrieved no other types of studies, we used the AGREE-II instrument only to assess methodological quality of the included guidelines (Appendix S10 in the **Online Supplementary Document**) [16]. All the guidelines were heavily marked down for not reporting on the rigour of evidence. This included not reporting systematic search methods, the development of criteria and methods for selecting and formulating evidence, the inclusion and exclusion criteria, a description of the strengths and limitations of the evidence, whether external experts had reviewed the guideline, and the process for updating the guidelines in the future. As per the AGREE II tool protocol, following the assessment, overall quality ratings were assigned from one (*i.e.* lowest possible quality) to seven (*i.e.* highest possible quality), and one of three recommendations was made (*i.e.* ‘for use’, ‘against use’, or ‘for use with modifications’). Based on the AGREE II tool, the guidelines from the USA [25], Chile [29], and Spain [27] were recommended for use and rated at the highest overall quality (Appendix S10 in the **Online Supplementary Document**).

We did not assess the quality of the simulation study as there is no standard tool.

## DISCUSSION

We found no studies from LMICs in our systematic review. Bed space was reported in only one guideline as 2.8 m^2^. Optimal space requirements for multi-cot areas were described by one simulation study [30] as 13.5 m^2^ per bed and the most recent USA guideline as 14 m^2^ per bed.[6] We located only two guidelines that described the space requirements in M-NICU as 20 m^2^ (inclusive of the bathroom) and 28 m^2^ (exclusive of the bathroom) [25,29]. There was no consistent reporting of definitions of bed and/or floor space amongst all the included studies.

The quality of papers varied. Although some of the lower AGREE-II scores of older guidelines could be partially explained by improvements in reporting standards and methodologies over time, the domains assessing the rigour of development and applicability were marked down across all guidelines, including those recently published. Most did not provide a search strategy, eligibility criteria, or details on the methods and research that supported their recommendations. They also did not use a standard checklist, such as the AGREE checklist [31].

Definitions of floor space between guidelines were difficult to interpret. For instance, the Chilean guideline recommended space for private single rooms; however, it was less clear whether these same space recommendations were also relevant to the multi-cot rooms [29]. As a result, we provided these space sizes as the dimensions of private rooms only. One of the biggest discrepancies was between the USA and Chile guidelines for couplet care rooms, 28 m^2^ (exclusive of the bathroom) and 20 m^2^ (inclusive of the bathroom), respectively. Although there are limited details to discern why these changes are so different, one aspect of the Chilean couplet care room is that it was intended for a much shorter stay, with newborns and their families staying in the room before and to support the discharge of their infants. As a result, it is likely that the couplet care rooms described in the Chile guidelines are not intended for the long-term care of infants. Overall, these issues have contributed to the inability to provide any summary statistics for space within NICUs.

Determining the optimal space requirements for SSN units is crucial not only for understanding the appropriate benchmarks for scaling up SSN service delivery in LMIC health facilities but also for broader implications in reducing overcrowding in NICUs. In turn, this protects from infections for the most vulnerable infants. However, this cannot be taken in isolation, as staffing and access to sinks for hygiene control are also important factors in minimising infections. For example, there is evidence to suggest that hospital-acquired infections increase when the sink-to-cot ratio is lower [32], and that an increase in bed space can reduce the cross-contamination of various bacteria [33,34]. Understanding additional SSN unit space requirements beyond just bed space, including dedicated hand hygiene facilities within patient zones, and sufficient overall environmental space to facilitate optimal workflow and cleaning, is crucial for effectively reducing infections in neonates, especially in resource-constrained settings.

We did not attempt to understand the functionality of space or how families perceive it. We aimed to provide evidence on optimal space requirements in NICUs, particularly level 2 health facilities. This work contributes to a larger body of knowledge that includes understanding the optimal number of beds and determining the necessary staff numbers for a NICU. For WHO experts to make further recommendations, it would be ideal to also understand space functionality and preferences of families, especially since we found no studies to support optimal space requirements for LMIC. Benchmarking optimal space for LMICs using HIC data, which is likely to recommend high optimal space requirements, is likely to be problematic due to issues surrounding the cost and staffing needed to create these spaces. In HIC countries, countries can afford the additional space and staffing when creating or redesigning NICU spaces. This is not always the case for LMIC, where optimal space needs to be considered in the context of available resources. It is possible that LMICs need to consider more innovative solutions to add to already existing structures, such as modular NICUs or incorporating additional tools, such as facility readiness or an updated assessment tool, to target facility-based maternal and neonatal infection prevention and control practices [35,36]. Additionally, the use of a simulation-based testing model, as provided by Hignett et al. [30], might offer a process for determining optimal SSN unit size when constraints already exist within the neonatal environment. This process could inform SSN unit redesign, providing LMICs with the optimal space, until such a time as a country can invest in additional space requirements.

Our systematic review had several limitations. We did not include grey literature as much of the grey literature in this space reported various hospital- and/or country-based guidelines on a SSN unit design, which largely reiterated what was in the published literature. For instance, according to the European guidelines [37], a multi-bed NICU space was a minimum of 18 m^2^ (inclusive of chair/bed), while in Australia [38], it was 17 m^2^ (inclusive of basin). We also did not include qualitative evidence, which provides insights into family values and preferences within NICUs. However, these data did not contribute to our aim. We also developed the search strategy within the team using Systematic Review Accelerator. Despite this, we conducted extensive database searches, including additional databases relevant to the review topic, such as CINAHL for nursing-related issues and the regional database LILACS, although we still found limited information.

## CONCLUSIONS

We found no studies of optimal space requirements for SSN unit in LMICs. With only the HIC country available for optimal space requirements, there is likely to be more context-specific estimates needed to support these countries. This would include more high-quality studies, especially simulation studies, to understand functionality and infection control. In addition, as countries await the resources to develop optimal SSN unit spaces, they could utilise tools to improve infection control as an effective alternative.

## Additional material


Online Supplementary Document
