# Supplementary material for: Determining space requirements for small and sick newborns and their mothers in health facilities: a systematic review
Source: J Glob Health. 2025 Oct 17;15:04313. doi: 10.7189/jogh.15.04313 (PMC12532444; doi:10.7189/jogh.15.04313)
Supplement: Online Supplementary Document [file jogh-15-04313-s001.pdf]

Supplement to: Strobel N, Whisson G, Swe D, Mehta R, Budrikis A, Edmond K. Determining space requirements for small and sick newborns and their mothers in health facilities: a systematic review. J Glob Health. 2025;15:04313.

Table of Contents

Appendix S1: PRISMA Checklist 2

Appendix S2: SWiM PRISMA extension 5

Appendix S3: Search strategies 6

Appendix S4: Example of data extraction form 9

Appendix S5: PRISMA Flow Diagram 11

Appendix S6: Excluded studies with reasons 12

Appendix S7: Characteristics of included guidelines 19

Appendix S8: Space requirements in a NICU 20

Appendix S9: Optimal space requirements for other NICU room types 22

Appendix S10: AGREE-II 23

Appendix S1: PRISMA Checklist

Section and Topic	Item #	Checklist item	Location where item is reported
**TITLE**			
Title	1	Identify the report as a systematic review.	1
**ABSTRACT**			
Abstract	2	See the PRISMA 2020 for Abstracts checklist.	2
**INTRODUCTION**			
Rationale	3	Describe the rationale for the review in the context of existing knowledge.	3-4
Objectives	4	Provide an explicit statement of the objective(s) or question(s) the review addresses.	4
**METHODS**			
Eligibility criteria	5	Specify the inclusion and exclusion criteria for the review and how studies were grouped for the syntheses.	4-5
Information sources	6	Specify all databases, registers, websites, organisations, reference lists and other sources searched or consulted to identify studies. Specify the date when each source was last searched or consulted.	5
Search strategy	7	Present the full search strategies for all databases, registers and websites, including any filters and limits used.	Appendix 3
Selection process	8	Specify the methods used to decide whether a study met the inclusion criteria of the review, including how many reviewers screened each record and each report retrieved, whether they worked independently, and if applicable, details of automation tools used in the process.	4-5
Data collection process	9	Specify the methods used to collect data from reports, including how many reviewers collected data from each report, whether they worked independently, any processes for obtaining or confirming data from study investigators, and if applicable, details of automation tools used in the process.	5-6
Data items	10a	List and define all outcomes for which data were sought. Specify whether all results that were compatible with each outcome domain in each study were sought (e.g. for all measures, time points, analyses), and if not, the methods used to decide which results to collect.	5
	10b	List and define all other variables for which data were sought (e.g. participant and intervention characteristics, funding sources). Describe any assumptions made about any missing or unclear information.	4, 5
Study risk of bias assessment	11	Specify the methods used to assess risk of bias in the included studies, including details of the tool(s) used, how many reviewers assessed each study and whether they worked independently, and if applicable, details of automation tools used in the process.	6
Effect measures	12	Specify for each outcome the effect measure(s) (e.g. risk ratio, mean difference) used in the synthesis or presentation of results.	NA

Section and Topic	Item #	Checklist item	Location where item is reported
Synthesis methods	13a	Describe the processes used to decide which studies were eligible for each synthesis (e.g. tabulating the study intervention characteristics and comparing against the planned groups for each synthesis (item #5)).	5-6
	13b	Describe any methods required to prepare the data for presentation or synthesis, such as handling of missing summary statistics, or data conversions.	NA
	13c	Describe any methods used to tabulate or visually display results of individual studies and syntheses.	6
	13d	Describe any methods used to synthesize results and provide a rationale for the choice(s). If meta-analysis was performed, describe the model(s), method(s) to identify the presence and extent of statistical heterogeneity, and software package(s) used.	6
	13e	Describe any methods used to explore possible causes of heterogeneity among study results (e.g. subgroup analysis, meta-regression).	NA
	13f	Describe any sensitivity analyses conducted to assess robustness of the synthesized results.	NA
Reporting bias assessment	14	Describe any methods used to assess risk of bias due to missing results in a synthesis (arising from reporting biases).	NA
Certainty assessment	15	Describe any methods used to assess certainty (or confidence) in the body of evidence for an outcome.	NA
**RESULTS**			
Study selection	16a	Describe the results of the search and selection process, from the number of records identified in the search to the number of studies included in the review, ideally using a flow diagram.	6, Appendix 5
	16b	Cite studies that might appear to meet the inclusion criteria, but which were excluded, and explain why they were excluded.	Appendix 6
Study characteristics	17	Cite each included study and present its characteristics.	6-7, Table 2
Risk of bias in studies	18	Present assessments of risk of bias for each included study.	8, Table 5
Results of individual studies	19	For all outcomes, present, for each study: (a) summary statistics for each group (where appropriate) and (b) an effect estimate and its precision (e.g. confidence/credible interval), ideally using structured tables or plots.	Table 3
Results of syntheses	20a	For each synthesis, briefly summarise the characteristics and risk of bias among contributing studies.	6-8
	20b	Present results of all statistical syntheses conducted. If meta-analysis was done, present for each the summary estimate and its precision (e.g. confidence/credible interval) and measures of statistical heterogeneity. If comparing groups, describe the direction of the effect.	NA
	20c	Present results of all investigations of possible causes of heterogeneity among study results.	NA

Section and Topic	Item #	Checklist item	Location where item is reported
	20d	Present results of all sensitivity analyses conducted to assess the robustness of the synthesized results.	NA
Reporting biases	21	Present assessments of risk of bias due to missing results (arising from reporting biases) for each synthesis assessed.	NA
Certainty of evidence	22	Present assessments of certainty (or confidence) in the body of evidence for each outcome assessed.	NA
**DISCUSSION**			
Discussion	23a	Provide a general interpretation of the results in the context of other evidence.	8-10
	23b	Discuss any limitations of the evidence included in the review.	10
	23c	Discuss any limitations of the review processes used.	10
	23d	Discuss implications of the results for practice, policy, and future research.	10
**OTHER INFORMATION**			
Registration and protocol	24a	Provide registration information for the review, including register name and registration number, or state that the review was not registered.	4
	24b	Indicate where the review protocol can be accessed, or state that a protocol was not prepared.	NA
	24c	Describe and explain any amendments to information provided at registration or in the protocol.	NA
Support	25	Describe sources of financial or non-financial support for the review, and the role of the funders or sponsors in the review.	10
Competing interests	26	Declare any competing interests of review authors.	11
Availability of data, code and other materials	27	Report which of the following are publicly available and where they can be found: template data collection forms; data extracted from included studies; data used for all analyses; analytic code; any other materials used in the review.	Appendix 4

Appendix S2: SWiM PRISMA extension

**SWiM reporting item**	**Item description**	**Page in manuscript where item is reported**
Methods		
**1** Grouping studies for synthesis	1a) Provide a description of, and rationale for, the groups used in the synthesis (e.g., groupings of populations, interventions, outcomes, study design)	3-4; 6
	1b) Detail and provide rationale for any changes made subsequent to the protocol in the groups used in the synthesis	NA
**2** Describe the standardised metric and transformation methods used	Describe the standardised metric for each outcome. Explain why the metric(s) was chosen, and describe any methods used to transform the intervention effects, as reported in the study, to the standardised metric, citing any methodological guidance consulted	5-6
**3** Describe the synthesis methods	Describe and justify the methods used to synthesise the effects for each outcome when it was not possible to undertake a meta-analysis of effect estimates	4-6
**4** Criteria used to prioritise results for summary and synthesis	Where applicable, provide the criteria used, with supporting justification, to select the particular studies, or a particular study, for the main synthesis or to draw conclusions from the synthesis (e.g., based on study design, risk of bias assessments, directness in relation to the review question)	NA
**SWiM reporting item**	**Item description**	**Page in manuscript where item is reported**
**5** Investigation of heterogeneity in reported effects	State the method(s) used to examine heterogeneity in reported effects when it was not possible to undertake a meta-analysis of effect estimates and its extensions to investigate heterogeneity	6
**6** Certainty of evidence	Describe the methods used to assess certainty of the synthesis findings	NA
**7** Data presentation methods	Describe the graphical and tabular methods used to present the effects (e.g., tables, forest plots, harvest plots).<br>Specify key study characteristics (e.g., study design, risk of bias) used to order the studies, in the text and any tables or graphs, clearly referencing the studies included	6
**8** Reporting results	For each comparison and outcome, provide a description of the synthesised findings, and the certainty of the findings. Describe the result in language that is consistent with the question the synthesis addresses, and indicate which studies contribute to the synthesis	6-8
Discussion		
**9** Limitations of the synthesis	Report the limitations of the synthesis methods used and/or the groupings used in the synthesis, and how these affect the conclusions that can be drawn in relation to the original review question	10

Appendix S3: Search strategies

Embase Classic+Embase <1947 to 2023 October 23>

No.	Query
1	(neo?nat* intensive care unit* or special care nurser* or NICU? or SCN? or neonat* ward).ti,ab,kf. or "neonatal intensive care unit".kw.
2	("Reproductive, Maternal, Newborn, Child, Primary care" or RMNCH or (EmOC or EmONC or "emergency obstetric care" or "Emergency Obstetric and Newborn Care") or EMC or SSNC or "special care" or "special care newborn unit*" or "special care nurser*" or "special care baby unit*" or "basic emergency obstetric care" or BEmOC or "comprehensive emergency obstetric care" or CeMOC).ti,ab.
3	newborn intensive care/ or neonatal intensive care unit/
4	((level 1 or level i or level one or level 2 or level ii or level two or level 3 or level iii or level three) adj3 (service? or hospital? or ward? or unit? or department? or dept? or centre? or center? or care)).ti,ab.
5	1 or 2 or 3 or 4
6	(design* adj2 (unit* or ward* or facilit* or "neo?nat* intensive care unit*" or "special care nurser*")).ti,ab.
7	(design* adj2 (NICU? or SCN? or SSNC? or EmOC or EmONC or RMNCH)).ti,ab.
8	(space* or spatial or layout* or arrange* or m2).ti,ab.
9	(((room or ward) adj2 (size? or layout or area)) or "minimum area" or "facility design" or "evidence?based design" or "NICU design").ti,ab.
10	("design evaluation" or "user-centered design").kw.
11	("standard? of care" adj2 (newborn? or neo?nat*)).ti,ab.
12	hospital design/ or "standards"/
13	((single? adj (bay or bed or room or cot)) or open?bay).ti,ab,kw.
14	6 or 7 or 8 or 9 or 10 or 11 or 12 or 13
15	5 and 14
16	(rat or rats or mouse or mice or rodent or rodents or swine or porcine or murine or sheep or lamb or lambs or ewe or ewes or pig or pigs or piglet or piglets or sow or sows or rabbit or rabbits or cat or cats or kitten or kittens or dog or dogs or puppy or puppies or monkey or monkeys or horse or horses or foal or foals or equine or calf or calves or cattle or heifer or heifers or hamster or hamsters or chicken or chickens or livestock or panda or pandas or buffalo$ or baboon$ or nonhuman or non-human).mp.
17	15 not 16

Ovid MEDLINE® ALL <1946 to October 24, 2023>

No.	Query
1	(neo?nat* intensive care unit* or special care nurser* or NICU? or SCN? or neonat* ward).ti,ab,kf. or "Intensive Care Units, Neonatal".kw.
2	("Reproductive, Maternal, Newborn, Child, Primary care" or RMNCH or (EmOC or EmONC or "emergency obstetric care" or "Emergency Obstetric and Newborn Care") or EMC or SSNC or "special care" or "special care newborn unit*" or "special care nurser*" or "special care baby unit*" or "basic emergency obstetric care" or BEmOC or "comprehensive emergency obstetric care" or CeMOC).ti,ab.
3	Intensive Care, Neonatal/ or "Perinatal Care"/ or Intensive Care Units, Neonatal/
4	((level 1 or level i or level one or level 2 or level ii or level two or level 3 or level iii or level three) adj3 (service? or hospital? or ward? or unit? or department? or dept? or centre? or center? or care)).ti,ab.
5	1 or 2 or 3 or 4
6	(design* adj2 (unit* or ward* or facilit* or "neo?nat* intensive care unit*" or "special care nurser*")).ti,ab.
7	(design* adj2 (NICU? or SCN? or SSNC? or EmOC or EmONC or RMNCH)).ti,ab.
8	(space* or spatial or layout* or arrange* or m2).ti,ab.
9	(((room or ward) adj2 (size? or layout or area)) or "minimum area" or "facility design" or "evidence?based design" or "NICU design").ti,ab.
10	("design evaluation" or "user-centered design" or "reference standards").kw.
11	("standard? of care" adj2 (newborn? or neo?nat*)).ti,ab.
12	"Hospital Design and Construction"/ or Evidence-Based Facility Design/
13	((single? adj (bay or bed or room or cot)) or open?bay).ti,ab,kw.
14	6 or 7 or 8 or 9 or 10 or 11 or 12 or 13
15	5 and 14
16	(rat or rats or mouse or mice or rodent or rodents or swine or porcine or murine or sheep or lamb or lambs or ewe or ewes or pig or pigs or piglet or piglets or sow or sows or rabbit or rabbits or cat or cats or kitten or kittens or dog or dogs or puppy or puppies or monkey or monkeys or horse or horses or foal or foals or equine or calf or calves or cattle or heifer or heifers or hamster or hamsters or chicken or chickens or livestock or panda or pandas or buffalo$ or baboon$ or nonhuman or non-human).mp.
17	15 not 16

Cochrane CENTRAL Date Run: 25/10/2023 05:30:28

No.	Query
#1	((neo?nat* NEXT (intensive care NEXT unit*)) or (special care NEXT nurser*) or NICU? or SCN? or (neonat* NEXT ward)):ti,ab,kw or "Intensive Care Units, Neonatal":kw
#2	("Reproductive, Maternal, Newborn, Child, Primary care" or RMNCH or (EmOC or EmONC or "emergency obstetric care" or "Emergency Obstetric and Newborn Care") or EMC or SSNC or "special care" or (special care newborn NEXT unit*) or (special care NEXT nurser*) or (special care baby NEXT unit*) or "basic emergency obstetric care" or BEmOC or "comprehensive emergency obstetric care" or CeMOC):ti,ab

#3	MeSH descriptor: [Intensive Care, Neonatal] explode all trees
#4	MeSH descriptor: [Intensive Care Units, Neonatal] explode all trees
#5	MeSH descriptor: [Perinatal Care] explode all trees
#6	((level 1 or level i or level one or level 2 or level ii or level two or level 3 or level iii or level three) NEAR/3 (service? or hospital? or ward? or unit? or department? or dept? or centre? or center? or care)):ti,ab
#7	#1 OR #2 OR #3 OR #4 OR #5 OR #6
#8	(design* NEAR/2 (unit* or ward* or facilit* or (neo?nat* NEXT (intensive care NEXT unit*)) or (special care NEXT nurser*))):ti,ab
#9	(design* NEAR/2 (NICU? or SCN? or SSNC? or EmOC or EmONC or RMNCH)):ti,ab
#10	(space* or spatial or layout* or arrange* or m2):ti,ab
#11	(((room or ward) NEAR/2 (size? or layout or area)) or "minimum area" or "facility design" or "evidence based design" or "NICU design"):ti,ab
#12	("design evaluation" or "user-centered design" or "reference standards"):kw
#13	((standard? NEAR/2 care) NEAR/2 (newborn? or neo?nat*)):ti,ab
#14	MeSH descriptor: [Hospital Design and Construction] explode all trees
#15	MeSH descriptor: [Evidence-Based Facility Design] explode all trees
#16	((single? NEXT (bay or bed or room or cot)) or open?bay):ti,ab,kw
#17	#8 OR #9 OR #10 OR #11 OR #12 OR #13 OR #14 OR #15 OR #16
#18	#7 AND #17
#19	(rat or rats or mouse or mice or rodent or rodents or swine or porcine or murine or sheep or lamb or lambs or ewe or ewes or pig or pigs or piglet or piglets or sow or sows or rabbit or rabbits or cat or cats or kitten or kittens or dog or dogs or puppy or puppies or monkey or monkeys or horse or horses or foal or foals or equine or calf or calves or cattle or heifer or heifers or hamster or hamsters or chicken or chickens or livestock or panda or pandas or buffalo$ or baboon$ or nonhuman or non-human)
#20	#18 NOT #19
#21	#18 NOT #19<br>Trials

CINAHL Ultimate Date run: Wednesday, October 25, 2023 5:14:17 AM

No.	Query
S1	TI (((neo#nat* N1 (intensive care unit*)) or (special care nurser*) or NICU* or SCN* or (neonat* ward))) OR AB (((neo#nat* N1 (intensive care unit*)) or (special care nurser*) or NICU* or SCN* or (neonat* ward)))
S2	TI (("Reproductive, Maternal, Newborn, Child, Primary care" or RMNCH or EmOC or EmONC or "emergency obstetric care" or "Emergency Obstetric and Newborn Care" or EMC or SSNC or "special care" or "special care newborn unit*" or "special care nurser*" or "special care baby unit*" or "basic emergency obstetric care" or BEmOC or "comprehensive emergency obstetric care" or CeMOC)) OR AB (("Reproductive, Maternal, Newborn, Child, Primary care" or RMNCH or EmOC or EmONC or "emergency obstetric care" or "Emergency Obstetric and Newborn Care" or EMC or SSNC or "special care" or "special care newborn unit*" or "special care nurser*" or "special care baby unit*" or "basic emergency obstetric care" or BEmOC or "comprehensive emergency obstetric care" or CeMOC))
S3	MH intensive care units, neonatal
S4	MH intensive care, neonatal
S5	MH perinatal care
S6	TI (((level 1 or level i or level one or level 2 or level ii or level two or level 3 or level iii or level three) W3 (service* or hospital* or ward* or unit* or department* or dept* or centre* or center* or care))) OR AB (((level 1 or level i or level one or level 2 or level ii or level two or level 3 or level iii or level three) W3 (service* or hospital* or ward* or unit* or department* or dept* or centre* or center* or care)))
S7	S1 OR S2 OR S3 OR S4 OR S5 OR S6
S8	TI ((design* W2 (unit* or ward* or facilit* or "neo#nat* intensive care unit*" or "special care nurser*"))) OR AB ((design* W2 (unit* or ward* or facilit* or "neo#nat* intensive care unit*" or "special care nurser*")))
S9	TI ((design* W2 (NICU* or SCN* or SSNC* or EmOC or EmONC or RMNCH))) OR AB ((design* W2 (NICU* or SCN* or SSNC* or EmOC or EmONC or RMNCH)))
S10	TI ((space* or spatial or layout* or arrange* or m2)) OR AB ((space* or spatial or layout* or arrange* or m2))
S11	TI ((((room or ward) W2 (size* or layout or area)) or "minimum area" or "facility design" or "evidence#based design" or "NICU design")) OR AB ((((room or ward) W2 (size* or layout or area)) or "minimum area" or "facility design" or "evidence#based design" or "NICU design"))
S12	MJ facility design
S13	TI (("standard* of care" W2 (newborn* or neo#nat*))) OR AB (("standard* of care" W2 (newborn* or neo#nat*)))
S14	TI (((single* W2 (bay or bed* or room* or cot*)) or open#bay)) OR AB (((single* W2 (bay or bed* or room* or cot*)) or open#bay))
S15	MJ hospital design or hospital architecture or built environment
S16	S8 OR S9 OR S10 OR S11 OR S12 OR S13 OR S14 OR S15
S17	S7 AND S16
S18	TI ((rat or rats or mouse or mice or rodent or rodents or swine or porcine or murine or sheep or lamb or lambs or ewe or ewes or pig or pigs or piglet or piglets or sow or sows or rabbit or rabbits or cat or cats or kitten or kittens or dog or dogs or puppy or puppies or monkey or monkeys or horse or horses or foal or foals or equine or calf or calves or cattle or heifer or heifers or hamster or hamsters or chicken or chickens or livestock or panda or pandas or buffalo or buffaloes or baboon or baboons or nonhuman or non-human)) OR AB ((rat or rats or mouse or mice or rodent or rodents or swine or porcine or murine or sheep or lamb or lambs or ewe or ewes or pig or pigs or piglet or piglets or sow or sows or rabbit or rabbits or cat or cats or kitten or kittens or dog or dogs or puppy or puppies or monkey or monkeys or horse or horses or foal or foals or equine or calf or calves or cattle or heifer or heifers or hamster or

	hamsters or chicken or chickens or livestock or panda or pandas or buffalo or buffaloes or baboon or baboons or nonhuman or non-human))
S19	S17 NOT S18

LILACS Date Run: 25/10/2023

No	Query
#1	(NICU* OR SCN*) OR (neonatal intensive care unit*) OR (special care nurser*) OR (EMOC or SSNC or EMC)
#2	(facility design) or (layout design) or (spatial planning) or (single room) or (open bay) or (room size) or (evidence based design) or (user centered design) or (standard of care)
#3	#1 AND #2

Appendix S4: Example of data extraction form

NOTE: we have only provided the data extraction form for data we collected and not all the fields from other designs such as experimental and observational

General information

First author family name:

Year of publication:

Title:

What is the type of paper?

1. Full article
2. Protocol
3. Unclear
4. Other

Characteristics of included studies
Study design

1. Simulation study
2. Guideline
3. Observational study:
4. Experimental study:
5. Other

Aim:

Year:

Country/ries:

NICU Level:

Hospital setting:

Evidence of recommendations:

Scenario/General concept

Notes/Additional information

Characteristics of results

Multi-bed infant rooms

Extract reported data for Minimum area (m2/ft2) and adjacent aisle.

Family space, storage space, staff space, charting space, supply storage are all Yes/No to indicate if they are reported.

	**Minimum area (m2)**	**Adjacent aisle**	**Family space**	**Storage space**	**Staff space**	**Charting space**	**Supply storage**
**Optimal space requirements**							

Couplet care rooms

Extract reported data for Minimum area (m2/ft2) and adjacent aisle.

Family space, storage space, staff space, charting space, supply storage are all Yes/No to indicate if they are reported.

	**Minimum area (m2)**	**Adjacent aisle**	**Family space**	**Storage space**	**Staff space**	**Charting space**	**Supply storage**
**Optimal space requirements**							

Other room types

Extract reported data for Minimum area (m2/ft2)

	**Private (single-family) rooms**	**Specialised infant care space or room**	**Delivery room**	**Isolation room**	**Resuscitation room**
**Optimal space requirements**					

Additional information

Simulation studies
NICU Level

1. NICU (Level I)
2. NICU (Level II)
3. NICU (Level III)
4. NICU (Unspecified)
5. SSNC
6. SCN
7. EmONC
8. Other

Scenario

Example: Functional experiment size - floor area of 4.13 m × 3.27 m (13.5 ft x 10.7 ft) marked with 0.1 m (0.3 ft) calibration lines

Recommendation

Example: 13.50 m2 (145.3 sq ft): width 4.13 m (13.5 ft) × length 3.27 m (10.7 ft) per single cot space

Notes/Additional information

Other information
Contact information
Funding information
Conflicts of interest

Appendix S5: PRISMA Flow Diagram

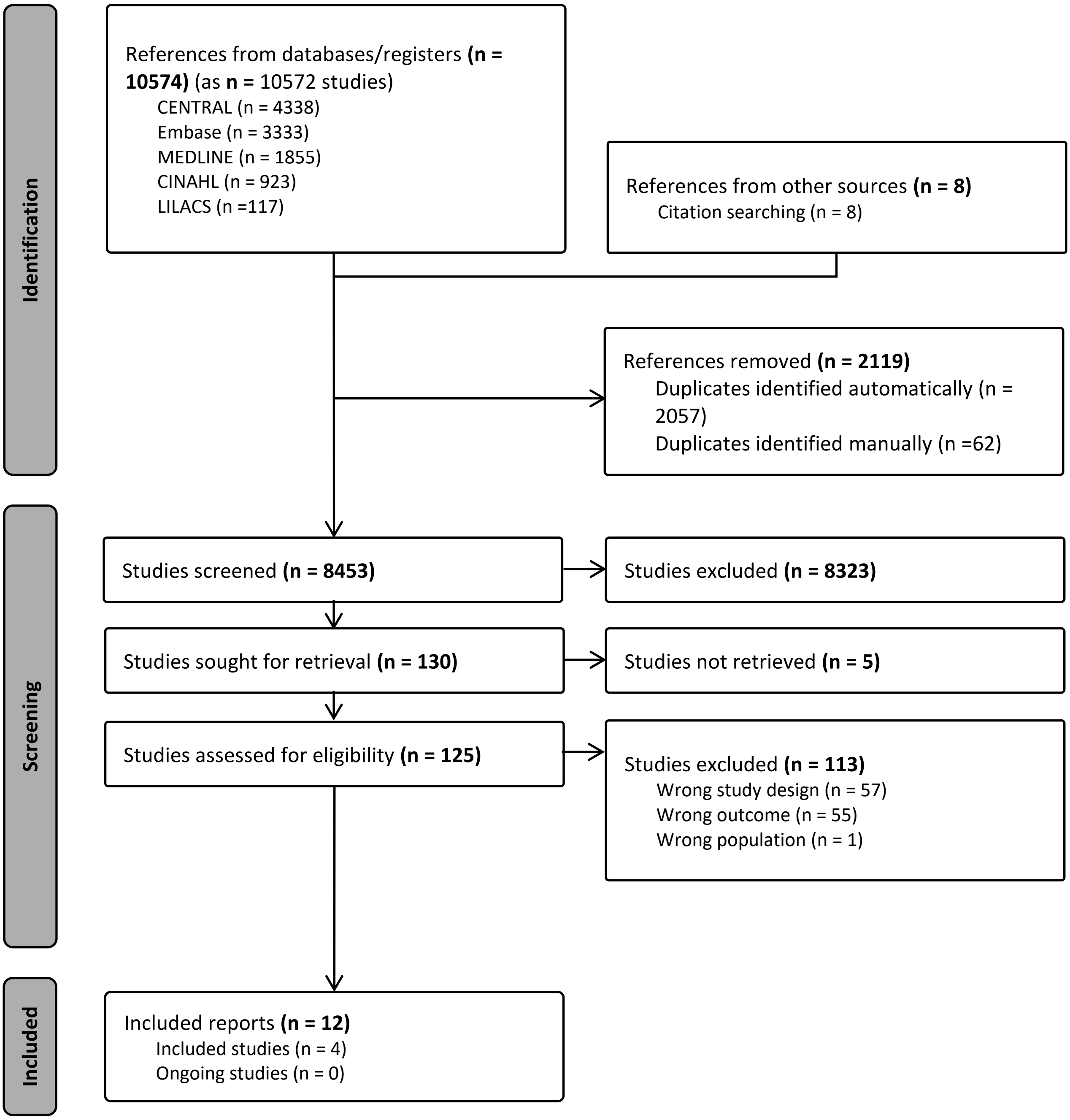

Appendix S6: Excluded studies with reasons

No.	Excluded citations	Reason
1	Aita, M., Robins, S., Charbonneau, L., Doray-Demers, P., & Feeley, N. (2021). Comparing light and noise levels before and after a NICU change of design. J Perinatol, 41(9), 2235-2243. doi:10.1038/s41372-021-01007-8	Wrong outcome
2	Al-Motlaq, M. A. (2018). Traditional open bay neonatal intensive care units can be redesigned to better suit family centered care application. Journal of Neonatal Nursing, 24(3), 159-162. doi:10.1016/j.jnn.2017.11.016	Wrong outcome
3	Alberman, E., Collingwood, J., & Pharoah, P. O. D. (1977). Arrangements for special and intensive care of the newborn. British Medical Journal, 2(6094), 1045-1047.	Wrong outcome
4	Altimier, L. (2001). High-tech, high-touch care. Nurs Manage, 32(7), 40-43. doi:10.1097/00006247-200107000-00018	Wrong study design
5	Altimier, L. B., Eichel, M., Warner, B., Tedeschi, L., & Brown, B. (2004). Developmental care: changing the NICU physically and behaviorally to promote patient outcomes and contain costs. Neonatal Intensive Care, 17(2), 35-39.	Wrong study design
6	Altimier, L. B., Eichel, M., Warner, B., Tedeschi, L., & Brown, B. (2005). Developmental care: changing the NICU physically and behaviorally to promote patient outcomes and contain costs. Neonatal Intensive Care, 18(4), 12-16.	Wrong outcome
7	Altimier, L., & Lutes, L. (2000). Changing units for changing times: the evolution of a NICU. Neonatal Intensive Care, 13(6), 23-27.	Wrong study design
8	Bailey, S., Taylor, A., & Kent, A. (2011). More space, Better quality care?Parents' perception of quality of care prior to and after neonatal unit relocation. Intensive Care Med, 37 Suppl 2, S428-S429. doi:10.1007/s00134-011-2387-x	Wrong study design
9	Basler, D. S. (1983). Principles of building a perinatal center. Clin Perinatol, 10(1), 9-30.	Wrong study design
10	Basnet, S., Adhikari, N., & Koirala, J. (2011). Challenges in setting up pediatric and neonatal intensive care units in a resource-limited country. Pediatrics, 128(4), e986-e992. doi:10.1542/peds.2010-3657	Wrong study design
11	Beck, S. A., Weis, J., Greisen, G., Andersen, M., & Zoffmann, V. (2009). Room for family-centered care -- a qualitative evaluation of a Neonatal Intensive Care Unit remodeling project. Journal of Neonatal Nursing, 15(3), 88-99.	Wrong outcome
12	Bender, G. J. (2011). In Situ Simulation for Systems Testing in Newly Constructed Perinatal Facilities. Semin Perinatol, 35(2), 80-83. doi:10.1053/j.semperi.2011.01.007	Wrong outcome
13	Bowie, B. H., Hall, R. B., Faulkner, J., & Anderson, B. (2003). Single-room infant care: future trends in special care nursery planning and design. Neonatal Network, 22(4), 27-34.	Wrong outcome
14	Broom, M., Kecskes, Z., Kildea, S., & Gardner, A. (2019). Exploring the Impact of a Dual Occupancy Neonatal Intensive Care Unit on Staff Workflow, Activity, and Their Perceptions. HERD, 12(2), 44-54. doi:10.1177/1937586718779360	Wrong outcome
15	Brown, P., & Taquino, L. T. (2001). Designing and delivering neonatal care in single rooms. The Journal of perinatal & neonatal nursing, 15(1), 68-83.	Wrong outcome
16	Carroll, S. (2022). A Unit Designed for the Neuroprotective Needs of Tiny Texans Part Two: Our Journey to the Ideal Small Baby NICU. Neonatal Intensive Care, 35(2), 30-32. Retrieved from https://nicmag.ca/pdf/NIC-35-2-Spring-2022-R19-web.pdf	Wrong study design
17	Carter, B. S., Carter, A., & Bennett, S. (2008). Families' views upon experiencing change in the neonatal intensive care unit environment:	Wrong outcome

	from the 'baby barn' to the private room. J Perinatol, 28(12), 827-829. doi:10.1038/jp.2008.102	
18	Carvajal de la Osa, J., Santana Gonzalez, J., Herrera Galan, M., Sanchez Grau, A., & Perez Almirall, I. (2020). Environmental magnetic field in a Neonatal Intensive Care Unit. A relevant verification. Arch Argent Pediatr, 118(3), E246-E251. doi:10.5546/aap.2020.eng.e246	Wrong outcome
19	Coston, A. D., & Aune, C. (2019). Reducing noise in the neonatal intensive care unit. Pediatrics, 144(2). doi:10.1542/peds.144.2-MeetingAbstract.154	Wrong study design
20	Coutts, S., Woldring, A., Pederson, A., De Salaberry, J., Osiovich, H., & Brotto, L. A. (2021). What is stopping us? An implementation science study of kangaroo care in British Columbia's neonatal intensive care units. BMC Pregnancy Childbirth, 21(1), 52. doi:10.1186/s12884-020-03488-5	Wrong study design
21	DellaPorta, K., Aforismo, D., & Butler-O'Hara, M. (1998). Co-bedding of twins in the neonatal intensive care unit. Pediatr Nurs, 24(6), 529-531.	Wrong study design
22	Denham, M. E., Bushehri, Y., & Lim, L. (2018). Through the Eyes of the User: Evaluating Neonatal Intensive Care Unit Design. HERD, 11(3), 49-65. doi:10.1177/1937586718761017	Wrong outcome
23	Domanico, R., Davis, D. K., Coleman, F., & Davis, B. O. (2011). Documenting the NICU design dilemma: Comparative patient progress in open-ward and single family room units. J Perinatol, 31(4), 281-288. doi:10.1038/jp.2010.120	Wrong outcome
24	Evans, M., Broom, M., & Abdel-Latif, M. E. (2019). Open Plan and Two Cot NICU Design: Comparing Neonatal Neurodevelopmental Outcomes. Journal of Paediatrics and Child Health, 55(S1), 74. doi:10.1111/jpc.14410_59	Wrong outcome
25	Fay, L., Real, K., Haynes, S., & Daneshvar, Z. (2023). Examining Efficiency in Open-Bay and Single-Family Room NICU Designs. Adv Neonatal Care, 23(4), 355-364. doi:10.1097/ANC.0000000000001058	Wrong outcome
26	Fischer, D., Schlosser, R. L., Kempf, V. A. J., Wichelhaus, T. A., Klingebiel, T., Philippi, S., ... Reinheimer, C. (2019). Overcrowding in a neonatal intermediate care unit: Impact on the incidence of multidrug-resistant gram-negative organisms. BMC Infect Dis, 19(1), 357. doi:10.1186/s12879-019-3981-8	Wrong outcome
27	Flacking, R., & Dykes, F. (2013). 'Being in a womb' or 'playing musical chairs': The impact of place and space on infant feeding in NICUs. BMC Pregnancy Childbirth, 13, 179. doi:10.1186/1471-2393-13-179	Wrong outcome
28	Flacking, R., Lehtonen, L., Thomson, G., Axelin, A., Ahlqvist, S., Moran, V. H., ... Dykes, F. (2012). Closeness and separation in neonatal intensive care. Acta Paediatr, 101(10), 1032-1037. doi:10.1111/j.1651-2227.2012.02787.x	Wrong outcome
29	Floyd, A. M. (2005). Challenging designs of neonatal intensive care units. Crit Care Nurse, 25(5), 59-66.	Wrong outcome
30	Form will fit function at new U-M Center. (1987). Michigan hospitals, 23(8), 13-18.	Wrong study design
31	Frayer, W. W. (1983). Neonatal intensive care unit renovation. The New York Hospital--Cornell Medical Center 1975-1976. Clin Perinatol, 10(1), 153-165.	Wrong outcome
32	Gluck, L. (1970). Design of a perinatal center. Pediatr Clin North Am, 17(4), 777-791. doi:10.1016/s0031-3955(16)32480-4	Wrong study design
33	Goldmann, D. A., Durbin Jr, W. A., & Freeman, J. (1981). Nosocomial infections in a neonatal intensive care unit. J Infect Dis, 144(5), 449-459. doi:10.1093/infdis/144.5.449	Wrong outcome
34	Goldstein, N. D., Tuttle, D., Tabb, L. P., Paul, D. A., Eppes, S. C., & Eppes, S. C. (2018). Spatial and environmental correlates of organism	Wrong outcome

	colonization and infection in the neonatal intensive care unit. J Perinatol, 38(5), 567-573. doi:10.1038/s41372-017-0019-1	
35	Grome, A., Papautsky, E. L., Crandall, B., & Greenberg, J. (2019). Application of Human Factors in Neonatal Intensive Care Unit Redesign. Adv Health Care Manag, 18. doi:10.1108/S1474-823120190000018004	Wrong outcome
36	Haiek, L. N., Nyqvist, K. H., & Mastrup, R. (2013). The neo-BFHI: Development of the expansion of the baby-friendly hospital initiative into neonatal care. Breastfeed Med, 8(Suppl 1), S2. doi:10.1089/bfm.2013.9982	Wrong study design
37	Harrell, J. W., & Moon, R. G. (2008). Designs for the delicate: a look at evolving NICU design standard. Health facilities management, 21(12), 45-48.	Wrong study design
38	Harris, D. D., Shepley, M. M., White, R. D., Kolberg, K. J. S., & Harrell, J. W. (2006). The impact of single family room design on patients and caregivers: Executive summary. Journal of Perinatology, 26(Suppl 3), S38-S48. doi:10.1038/sj.jp.7211583	Wrong study design
39	Hart, M. C. (1983). The Phoenix Perinatal Center. Clin Perinatol, 10(1), 95-108.	Wrong study design
40	Holzman, I. R. (1987). "Doctor, fix this building.". American journal of perinatology, 4(1), 12-15. doi:10.1055/s-2007-999729	Wrong study design
41	Howell, L. J. (2013). The Garbose Family Special Delivery Unit: a new paradigm for maternal-fetal and neonatal care. Semin Pediatr Surg, 22(1), 3-9. doi:10.1053/j.sempedsurg.2012.10.002	Wrong outcome
42	Jansen, S., Berkhout, R. J. M., Te Pas, A. B., Steggerda, S. J., de Vries, L. S., Schalij-Delfos, N., ... Bekker, V. (2022). Comparison of neonatal morbidity and mortality between single-room and open-bay care: a retrospective cohort study. Arch Dis Child Fetal Neonatal Ed, 107(6), 611-616. doi:10.1136/archdischild-2021-323310	Wrong outcome
43	Jung, A. L., Kochenour, N. K., & Bose, C. L. (1983). The University of Utah Perinatal Center. An innovative design. Clin Perinatol, 10(1), 109-126.	Wrong study design
44	Korones, S. B. (1983). Evolution of nursery design and function. The Memphis story. Clin Perinatol, 10(1), 127-140.	Wrong study design
45	Kuhn, P., Sizun, J., Casper, C., Allen, A., Audeoud, F., Bouvard, C., ... Zores, C. (2018). Recommendations on the environment for hospitalised newborn infants from the French neonatal society: rationale, methods and first recommendation on neonatal intensive care unit design. Acta Paediatr, 107(11), 1860-1866. doi:10.1111/apa.14501	Wrong study design
46	Kuschel, C. A., & Roy, R. N. (2005). Who's got what? A benchmarking exercise for tertiary neonatal units. Journal of Paediatrics and Child Health, 41(12), 635-639.	Wrong study design
47	Larson, E., Hargiss, C. O., & Dyk, L. (1985). Effect of an expanded physical facility on nosocomial infections in a neonatal intensive care unit. American Journal of Infection Control, 13(1), 16-20. doi:10.1016/0196-6553(85)90004-5	Wrong outcome
48	Lee, K. (1984). Organisation of special and intensive care facilities for babies. Yonsei Med J, 25(1), 69-95. doi:10.3349/ymj.1984.25.1.69	Wrong study design
49	Lehtonen, L., Lee, S. K., Kusuda, S., Lui, K., Norman, M., Bassler, D., ... Shah, P. S. (2020). Family Rooms in Neonatal Intensive Care Units and Neonatal Outcomes: An International Survey and Linked Cohort Study. J Pediatr, 226, 112-117.e114. doi:10.1016/j.jpeds.2020.06.009	Wrong outcome
50	Leslie, A., & Kenner, C. (2010). Upgrading and extending existing neonatal intensive care facilities at Life Westville Hospital, Kwazulu-Natal, South Africa. Newborn and Infant Nursing Reviews, 10(2), 73-77. doi:10.1053/j.nainr.2010.03.005	Wrong study design
51	Li, Q. F., Xu, H., Ni, X. P., Lin, R., Jin, H., Wei, L. Y., ... Wu, B. (2017). Impact of relocation and environmental cleaning on reducing the	Wrong outcome

	incidence of healthcare-associated infection in NICU. World J Pediatr, 13(3), 217-221. doi:10.1007/s12519-017-0001-1	
52	Liew, E., & Cane, C. (2015). Individualised care rooms: The future of neonatal care. Archives of Disease in Childhood, 100(Suppl 3), A259-A260. doi:10.1136/archdischild-2015-308599.521	Wrong study design
53	Lindacher, V., Altebaeumer, P., Marlow, N., Matthaeus, V., Straszewski, I. N., Thiele, N., ... Mader, S. (2021). European Standards of Care for Newborn Health-A project protocol. Acta Paediatr, 110(5), 1433-1438. doi:10.1111/apa.15712	Wrong study design
54	Little babies, big dreams: Meriter nurses help drive new NICU design. (2007). Nursingmatters, 18(5), 20-21.	Wrong study design
55	Machry, H., White, R., & Barton, S. A. (2021). Gravens By Design: The Case of a NICU with Single-family Rooms: Design Recommendations to Support Family Engagement Behaviors. Neonatology Today, 16(9), 34-39. doi:10.51362/neonatology.today/202191693439	Wrong study design
56	Malhotra, S., Zodpey, S. P., Vidyasagaran, A. L., Sharma, K., Raj, S. S., Neogi, S. B., ... Saraf, A. (2014). Assessment of essential newborn care services in secondary-level facilities from two districts of India. J Health Popul Nutr, 32(1), 130-141.	Wrong outcome
57	Marshall-Baker, A. (2006). Human and environmental health: Sustainable design for the NICU. Journal of Perinatology, 26(Suppl 3), S31-S33. doi:10.1038/sj.jp.7211592	Wrong outcome
58	Martin, G. I. (2003). Recommended standards for newborn ICU design. J Perinatol, 23(SUPPL. 1), S3. doi:10.1038/sj.jp.7210833	Wrong study design
59	Martin, G. I. (2006). Recommended standards for newborn intensive care unit design. J Perinatol, 26(S3), S1. doi:10.1038/sj.jp.7211582	Wrong study design
60	McCoy, M., Makkar, A., Foulks, A., & Legako, E. (2014). Establishing level II neonatal services in southwestern Oklahoma. J Okla State Med Assoc, 107(9-10), 493-496.	Wrong study design
61	Medvedev, M. M., Tumukunde, V., Kirabo-Nagemi, C., Greco, G., Mambule, I., Katumba, K., ... Lawn, J. E. (2023). Process and costs for readiness to safely implement immediate kangaroo mother care: a mixed methods evaluation from the OMWaNA trial at five hospitals in Uganda. BMC Health Serv Res, 23(1), 613. doi:10.1186/s12913-023-09624-z	Wrong outcome
62	Milford, C. A., Zapalo, B. J., & Davis, G. (2008). Transition to an individual-room NICU design: process and outcome measures. Neonatal Netw, 27(5), 299-305. doi:10.1891/0730-0832.27.5.299	Wrong study design
63	Mitsunobu, T., & Misao, H. (2021). An Investigation of the Bed Area Pre-Neonate as a Risk Factor for MRSA in NICU. Association for Professionals in Infection Control and Epidemiology Annual Conference (Virtual), 28-30 June, 2021. American Journal of Infection Control, 49(6), S15-S15. doi:10.1016/j.ajic.2021.04.060	Wrong study design
64	Murphy, J., & Hodson, W. A. (1974). Neonatal intensive care. Design and function of a special unit... part 2. Postgraduate Medicine, 56, 65-70.	Wrong outcome
65	Nejad, M. R., Heidarzadeh, M., Mohagheghi, P., Akrami, F., Almasi-Hashiani, A., & Eskandari, Z. (2017). Assessment of physical environment of Iran's neonatal tertiary care centers from the perspective of the neonatal individualized developmental care. Iranian Journal of Neonatology, 8(4), 20-25. doi:10.22038/ijn.2017.21258.1240	Wrong outcome
66	Northern Neonatal Network. (1993). Requirements for neonatal cots. Archives of Disease in Childhood, 68(5 Spec No), 544-549.	Wrong outcome
67	Nubel, U., Nachtnebel, M., Falkenhorst, G., Benzler, J., Hecht, J., Kube, M., ... Eckmanns, T. (2013). MRSA transmission on a neonatal intensive care unit: epidemiological and genome-based phylogenetic analyses. PloS one, 8(1), e54898. doi:10.1371/journal.pone.0054898	Wrong outcome

68	Obeidat, B., Younis, M. B., Al-Shlool, E., & Alzouby, A. (2022). A Study of Workspace Design Characteristics Exemplified by Nurses' Satisfaction Within Three Intensive Care Units in a University Hospital. HERD, 15(2), 63-78. doi:10.1177/19375867211055731	Wrong population
69	Panknin, H. T. (2013). [Neonatal intensive care units: structural design effects patient care and coworker satisfaction]. Kinderkrankenschwester, 32(6), 228-229.	Wrong outcome
70	Parry, G. J., Tucker, J. S., Tarnow-Mordi, W. O., & U. K. Neonatal Staffing Study Group. (2005). Relationship between probable nosocomial bacteraemia and organisational and structural factors in UK neonatal intensive care units. Qual Saf Health Care, 14(4), 264-269. doi:10.1136/qshc.2004.012690	Wrong outcome
71	Pérez-Muñuzuri, A., Boix, H., Sánchez-Redondo, M. D., Cernada, M., Espinosa-Fernández, M. G., González-Pacheco, N., ... Luna, M. S. (2023). Niveles asistenciales en las unidades neonatales en España: Una visión actualizada para una nueva realidad. Anales de Pediatría, 98(4), 301-307.	Wrong outcome
72	Pineda, R. G., Neil, J., Dierker, D., Smyser, C. D., Wallendorf, M., Kidokoro, H., ... Inder, T. (2014). Alterations in brain structure and neurodevelopmental outcome in preterm infants hospitalized in different neonatal intensive care unit environments. J Pediatr, 164(1), 52-60.e52. doi:10.1016/j.jpeds.2013.08.047	Wrong study design
73	Pineda, R. G., Stransky, K. E., Rogers, C., Duncan, M. H., Smith, G. C., Neil, J., & Inder, T. (2012). The single-patient room in the NICU: Maternal and family effects. J Perinatol, 32(7), 545-551. doi:10.1038/jp.2011.144	Wrong outcome
74	Pineda, R., Kati, K., Breault, C. C., Rogers, E. E., Mack, W. J., & Fernandez-Fernandez, A. (2023). NICUs in the US: levels of acuity, number of beds, and relationships to population factors. J Perinatol, 43(6), 796-805. doi:10.1038/s41372-023-01693-6	Wrong outcome
75	Salihoglu, O., Akkus, C. H., & Hatipoglu, S. (2011). Recommended standards for the newborn intensive care unit (NICU). Medical Journal of Bakirkoy, 7(2), 45-51. doi:10.5350/btdmjb201107201	Wrong study design
76	Saucier, R. (2010). Design standardization in the private neonatal intensive care unit room. Newborn & Infant Nursing Reviews, 10(2), 92-96. doi:10.1053/j.nainr.2010.03.008	Wrong outcome
77	Scallon, S. K., Lajoie, J., Ness, P. J., Habib, M., Mahatma, G., Tauber, K. A., & Barry, G. P. (2022). Retinopathy of prematurity rates after transition from multi-bed to single-bed neonatal intensive care unit. Journal of AAPOS, 26(4), e55. doi:10.1016/j.jaapos.2022.08.207	Wrong study design
78	Shepley, M. M. (2002). Predesign and postoccupancy analysis of staff behavior in a neonatal intensive care unit. Children's Health Care, 31(3), 237-253. doi:10.1207/s15326888chc3103_5	Wrong outcome
79	Shepley, M. M., Smith, J. A., Sadler, B. L., & White, R. D. (2014). The business case for building better neonatal intensive care units. J Perinatol, 34(11), 811-815. doi:10.1038/jp.2014.174	Wrong outcome
80	Sheridan, J. F. (1983). The typical perinatal center. An overview of perinatal health services in the United States. Clin Perinatol, 10(1), 31-47.	Wrong study design
81	Shuman, C. J., Morgan, M., & Vance, A. (2023). Integrating Neonatal Intensive Care Into a Family Birth Center: Describing the Integrated NICU (I-NIC). J Perinat Neonatal Nurs, 39(1), 64-73. doi:10.1097/JPN.0000000000000759	Wrong outcome
82	Smith, J. (1994). Clinically speaking: issues in designing the NICU. Mo Nurse, 63(6), 5. Retrieved from https://www.ncbi.nlm.nih.gov/pubmed/7731450	Wrong study design
83	Smith, J. (2022). Gravens By Design: Common Questions for Designing Today's State-of-the-Art NICU. Neonatology Today, 17(5), 59-63. doi:10.51362/neonatology.today/20221755963	Wrong study design

84	Song, I. G., Shin, S. H., & Kim, H. S. (2018). Improved Regional Disparities in Neonatal Care by Government-led Policies in Korea. J Korean Med Sci, 33(6), e43. doi:10.3346/jkms.2018.33.e43	Wrong study design
85	Special care units have special design requirements. (1972). Modern hospital, 118(3), 105-107.	Wrong study design
86	Stanojevic, M. (2004). Equipement of maternity wards in Croatia in 2003: Neonatology. Gynaecologia et Perinatologia, 13(Suppl 3), 36-44.	Wrong study design
87	Stelwagen, M., Westmaas, A., Kempen van, A., Blees, Y., & Scheele, F. (2018). A new infrastructure for patient empowerment through Family Integrated Obstetric and Neonatal Healthcare in Single Family Rooms. A case study. International Journal of Integrated Care, 18(S2), 1-2. doi:10.5334/ijic.s2356	Wrong study design
88	Stevens, D. C., Helseth, C. C., Akram Khan, M., Munson, D. P., & Reid, E. J. (2011). A Comparison of Parent Satisfaction in an Open-Bay and Single-Family Room Neonatal Intensive Care Unit. HERD, 4(3), 110-123. doi:10.1177/193758671100400309	Wrong study design
89	Stevens, D. C., Helseth, C. C., Thompson, P. A., Pottala, J. V., Khan, M. A., & Munson, D. P. (2012). A Comprehensive Comparison of Open-Bay and Single-Family-Room Neonatal Intensive Care Units at Sanford Children's Hospital. HERD, 5(4), 23-39.	Wrong outcome
90	Stevens, D. C., Thompson, P. A., Helseth, C. C., Hsu, B., Khan, M. A., & Munson, D. P. (2014). A comparison of the direct cost of care in an open-bay and single-family room NICU. J Perinatol, 34(11), 830-835. doi:10.1038/jp.2014.178	Wrong outcome
91	Stevens, D. C., Thompson, P. A., Helseth, C. C., Pottala, J. V., Khan, M. A., & Munson, D. P. (2011). A comparison of outcomes of care in an open-bay and single-family room neonatal intensive care facility. Journal of Neonatal-Perinatal Medicine, 4(3), 189-200. doi:10.3233/npm-2011-2762	Wrong outcome
92	Stichler, J. F. (2012). The new standard: Single family room design. J Nurs Adm, 42(10), 447-450. doi:10.1097/NNA.0b013e31826a1cd2	Wrong study design
93	Terrin, G., Conte, F., Scipione, A., Aleandri, V., Di Chiara, M., Bacchio, E., ... De Curtis, M. (2016). New architectural design of delivery room reduces morbidity in preterm neonates: A prospective cohort study. BMC Pregnancy Childbirth, 16(1), 63. doi:10.1186/s12884-016-0849-4	Wrong outcome
94	The Newborn Intensive Care Unit. (2003). Journal of Perinatology, 23(Suppl 1), S8. doi:10.1038/sj.jp.7210837	Wrong study design
95	Thompson, H., Legorreta, K., Maher, M. A., & Lavin, M. M. (2016). Planning, Designing, Building, and Moving a Large Volume Maternity Service to a New Labor and Birth Unit. MCN Am J Matern Child Nurs, 41(6), 322-331. doi:10.1097/NMC.0000000000000276	Wrong study design
96	Trujillo, J. L., Avino, A. M., & Millan, C. L. (2017). User Evaluation of Neonatology Ward Design. HERD, 10(2), 23-48. doi:10.1177/1937586716641275	Wrong outcome
97	Tudehope, D. I., Lee, W., Harris, F., & Addison, C. (1989). Cost-analysis of neonatal intensive and special care. Australian paediatric journal, 25(2), 61-65.	Wrong outcome
98	Van Enk, R. A., & Steinberg, F. (2011). Comparison of private room with multiple-bed ward neonatal intensive care unit. HERD, 5(1), 52-63. doi:10.1177/193758671100500105	Wrong outcome
99	Vestal, R. (1999). Building blocks: how one hospital designed the core components of a new NICU. AWHONN Lifelines, 3(3), 37-39.	Wrong study design
100	Vidyasagar, D., & Work, B. (1983). The perinatal unit at the University of Illinois Hospital at Chicago. Clinics in perinatology, 10(1), 87-94.	Wrong study design
101	Villeneuve, E., Landa, P., Allen, M., Spencer, A., Prosser, S., Gibson, A., ... Pitt, M. (2018). Health Services and Delivery Research. A	Wrong outcome

	framework to address key issues of neonatal service configuration in England: the NeoNet multimethods study. doi:10.3310/hsdr06350	
102	Von Dolinger de Brito, D., de Almeida Silva, H., Jose Oliveira, E., Arantes, A., Abdallah, V. O. S., Tannus Jorge, M., & Gontijo Filho, P. P. (2007). Effect of neonatal intensive care unit environment on the incidence of hospital-acquired infection in neonates. J Hosp Infect, 65(4), 314-318. doi:10.1016/j.jhin.2006.01.038	Wrong outcome
103	Weber, D. O. (1996). Life-enhancing design. Healthc Forum J, 39(2), 39-49.	Wrong study design
104	Wei, L., He, S. S., & Zhang, X. H. (2023). Impact of the environmental layout of the neonatal intensive care unit on clinical outcomes and neurological development in very/extremely preterm infants. Zhongguo Dang Dai Er Ke Za Zhi, 25(8), 812-817. doi:10.7499/j.issn.1008-8830.2302060	Wrong outcome
105	White, R. (2014). NICU design. Archives of Disease in Childhood, 99(S2), A10. doi:10.1136/archdischild-2014-307384.28	Wrong study design
106	White, R. D. (2010). Single-Family Room Design in the Neonatal Intensive Care Unit-Challenges and Opportunities. Newborn Infant Nurs Rev, 10(2), 83-86. doi:10.1053/j.nainr.2010.03.011	Wrong study design
107	White, R. D. (2011). The Newborn Intensive Care Unit Environment of Care: How We Got Here, Where We're Headed, and Why. Semin Perinatol, 35(1), 2-7. doi:10.1053/j.semperi.2010.10.002	Wrong study design
108	White, R. D. (2013). Recommended NICU design standards and the physical environment of the NICU. J Perinatol, 33(Suppl 1), S1. doi:10.1038/jp.2013.9	Wrong study design
109	White, R. D. (2016). The next big ideas in NICU design. J Perinatol, 36(4), 259-262. doi:10.1038/jp.2016.6	Wrong study design
110	White, R. D. (2022). Gravens By Design: Addressing Challenges to Fully Incorporating Families into the NICU Care Team. Neonatology Today, 17(10), 61-62. doi:10.51362//neonatology.today/202217106162	Wrong study design
111	White, R., & Graven, S. M. (2001). New concepts, science, experiences drive innovative designs: the changing face of the newborn ICU. Advances in Family-Centered Care, 7(1), 7-10.	Wrong study design
112	White, R., & Whitman, T. (1992). Design of ICUs. Pediatrics, 89(6), 1267.	Wrong study design
113	Xu, T., Yue, Q., Wang, Y., Murray, J., & Sobel, H. (2018). Childbirth and Early Newborn Care Practices in 4 Provinces in China: A Comparison With WHO Recommendations. Glob Health Sci Pract, 6(3), 565-573. doi:10.9745/GHSP-D-18-00017	Wrong study design

Appendix S7: Characteristics of included guidelines

Study ID	Aim	Country	NICU Setting	Evidence of recommendation
Martin 1999(1)	The purpose of this committee is to provide healthcare professionals, architects, interior designers, state healthcare facility regulators, and others involved in the planning of NICUs with a comprehensive set of standards.	United States	Newborn intensive care	Expert consensus committee
Martin 2003(2)	Provide the basis for a consistent set of standards that can be adopted by all states and endorsed by appropriate national organizations, and support the international field	United States	NICU (unspecified)	Expert consensus committee
Blanco Bravo 2004(3)	Provide levels of care and minimum recommendations for neonatal care	Spain	NICU Levels I, II, III	Expert committee consensus
White 2006(4)	The purpose of this committee is to provide healthcare professionals, architects, interior designers, state healthcare facility regulators, and others involved in the planning of NICUs with a comprehensive set of standards.	United States	NICU (unspecified)	Expert consensus committee
White 2007(5)	To provide the basis for a consistent set of standards that can be used by all states and endorsed by appropriate national organizations, and support international arena.	United States	NICU Level III	Expert consensus committee recommendations
Novoa 2009(6)	The Board of Directors of the Neonatology Branch of the Chilean Pediatric Society, with the collaboration of different experts from National Hospital Centers, proposes the following "Recommendations on the Organization, Characteristics and Functioning of Neonatology Services or Units" to serve as guidance in Neonatal Management.	Chile	NICU Levels I, II, III	Published literature*, Expert consensus committee
White 2013(7)	The purpose of this committee is to provide healthcare professionals, architects, interior designers, state healthcare facility regulators, and others involved in the planning of NICUs with a comprehensive set of standards.	United States	NICU Level III	Expert consensus committee
White 2020(8)	The purpose of this committee is to provide healthcare professionals, architects, interior designers, state healthcare facility regulators, and others involved in the planning of NICUs with a comprehensive set of standards.	United States	NICU (unspecified)	Expert committee consensus

NICU, neonatal intensive care unit; *It is unclear whether this evidence was used to create the recommendation for space.

Appendix S8: Space requirements in a NICU

Author Year	Bed size and/or minimum area	Adjacent aisle	Family physical space	Family storage space	Staff space	Charting space	Equipment and supply storage	Bed size and/or minimum area	Adjacent aisle	Family physical space	Family storage space	Staff space	Charting space	Equipment and supply storage
	Optimal space requirements for multi-bed infant rooms							Optimal space requirements for couplet care rooms						
Martin 1999(1)	Bed size intensive care: 2.8 m^2 (30 ft^2)<br><br>11.2 m^2 (120 ft^2) clear floor space^ excluding sinks and isles	1 m (3 ft)	Yes	Yes	Yes	Yes	Yes	-	-	-	-	-	-	-
Martin 2003(2)	Bed size intensive care: 2.8 m^2 (30 ft^2)<br><br>11.2 m^2 (120 ft^2) clear floor space^ excluding sinks and isles	1.2 m (4 ft) [2.4 m (8 ft) between cots]	Yes	Yes	Yes	Yes	Yes	-	-	-	-	-	-	-
Blanco Bravo 2004(3)	Bed size: NR<br><br>Minimum surface area (no other details)<br>Basic care: 1.5-2 m^2 (16-21.5 ft^2)<br>Special care: 4-5 m^2 (43-54 ft^2)<br>Intensive care: 9-11 m^2 (97-118.5 ft^2)	-	Yes	Yes	Yes	-	Yes	-	-	Yes	-	Yes	-	Yes
White 2006(4)	Bed size intensive care: 2.8 m^2 (30 ft^2)<br><br>11.2 m^2 (120 ft^2) clear floor space^ excluding sinks and isles	1.2 m (4 ft) [2.4 m (8 ft) between cots]	Yes	Yes	Yes	Yes	Yes	-	-	-	-	-	-	-
White 2007(5)	Bed size intensive care: 2.8 m^2 (30 ft^2)<br><br>11.2 m^2 (120 ft^2) clear floor space^ excluding sinks and isles	1.2 m (4 ft) [2.4 m (8 ft) between cots]	Yes	Yes	Yes	Yes	Yes	-	-	-	-	-	-	-

Novoa 2009(6)	Bed size: NR<br><br>Basic care: NR<br>Intermediate care: 4-5 m^2 (43-54 ft^2)<br>NICU: 9-11 m^2 (96.9-118.4 ft^2)<br>(excluding sinks and aisles)	Basic care: 1 m (3 ft) per side<br>Intermediate care: 1.2 m (4 ft) per side<br>NICU: 1.5 m (5 ft) per side	Yes	Yes	Yes	Yes	Yes	-	-	-	-	-	-	-
White 2013(7)	Bed size intensive care: 2.8 m^2 (30 ft^2)<br><br>11.2 m^2 (120 ft^2) clear floor space^ excluding sinks and isles	1.2 m (4 ft) [2.4 m (8 ft) between cots]	Yes	Yes	Yes	Yes	Yes	-	-	-	-	-	-	-
White 2020(8)	Bed size intensive care: 2.8 m^2 (30 ft^2)<br><br>14 m^2 (150 ft^2) clear floor space^ excluding sinks and isles	1.2 m (4 ft) [2.4 m (8 ft) between cots]	Yes	Yes	Yes	Yes	Yes	14 m^2 (150 ft^2) clear floor space^ for the NICU infant and 14 m^2 (150 ft^2) for the mother = 28 m^2 (300 ft^2)	2.4 m (8 ft) between beds	Yes	Yes	Yes	Yes	Yes

^Clear floor space is defined as the space available for functional use and excludes other defined spaces such as plumbing fixtures, anterooms, vestibules, toilet rooms, closets, lockers, wardrobes, fixed-based cabinets, and wall-hung counters; NR: not reported

Appendix S9: Optimal space requirements for other NICU room types

Author Year	Private SFRs	Additional spaces such as resuscitation or isolation room
Martin 1999(1)	-	Isolation room: 14 m^2 (150 ft^2) clear floor space^<br><br>Intensive care infant beds: may require 14 m^2 (150 ft^2) per infant to provide adequate space for equipment and families; those in chronic care areas may require more.
Martin 2003(2)	Minimum 14 m^2 (150 ft^2) includes space for equipment and families, those in chronic care areas may require more space	Isolation room: 14 m^2 (150 ft^2)
Blanco Bravo 2004(3)	-	Resuscitation station: Surface in specific area:12-15 m^2(129-162 ft^2)<br>Delivery/operating room:3-4 m^2 (32-43 ft^2)
White 2006(4)	Minimum 14 m^2 (150 ft^2) including equipment and families	Isolation room: 14 m^2 (150 ft^2) clear floor space^
White 2007(5)	Minimum size 14 m^2 (150 ft^2) clear floor space^	Isolation room: 14 m^2 (150 ft^2) clear floor space^<br>Resuscitation station: 3.7 m^2 (40 ft^2) clear floor space^<br>Operative delivery rooms: 7.5 m^2 (80 ft^2)
Novoa 2009(6)	-	Isolation room: minimum 12 m^2 (129 ft^2)<br>Lactation room: 2 m^2 (21.5 ft^2)
White 2013(7)	Minimum area 15.3 m^2 (165 ft^2) clear floor space^	Isolation room: 14 m^2 (150 ft^2) clear floor space^<br>Resuscitation station: 3.7 m^2 (40 ft^2) clear floor space^<br>Operative delivery rooms: 7.5 m^2 (80 ft^2)
Gracia 2013(9)	-	Delivery room/operating room including resuscitation: 3-4 m^2 (32-43 ft^2)
White 2020(8)	Minimum area 16.7 m^2 (180 ft^2) clear floor space^	Isolation room: 16.7 m^2 (180 ft^2) clear floor space<br>Resuscitation station: 3.7 m^2 (40 ft^2) clear floor space^<br>Operative delivery rooms: 7.5 m^2 (80 ft^2)<br>Specialised procedure space (e.g. ECMO): 33.5 m^2 (360 ft^2) clear floor area^
Bajaña 2021(10)	Level I: 12-14 m^2 (129-150 ft^2)<br>Level II: 14-16 m^2 (150-172 ft^2)<br>Level III/IV: 16-20 m^2 (172- 215 ft^2)*	Isolation room: 20 - 24 m^2 (215-258 ft^2)<br>Emergency surgical procedure room: 20 m^2 (215 ft^2)
Altimier 2023(11)	Minimum size 16.7 m^2 (180 ft^2) clear floor space^	Isolation room: 16.7 m^2 (180 ft^2)<br>Resuscitation station: 3.7 m^2 (40 ft^2) clear floor space^<br>Operative delivery rooms: 7.5 m^2 (80 ft^2)<br>Specialised beds (e.g. ECMO): 28 m^2 (300 ft^2) clear floor area^ SFRs<br>Specialised beds (e.g. ECMO): 21 m^2 (225 ft^2) clear floor space^

m: meters; ft: feet; SFRs: single family rooms
*inclusive of crib, monitoring and treatment equipment, ensure sufficient space for the prolonged stay of the family with comfortable furniture (e.g. recliners) and the interaction of parents with the newborn^Clear floor space is defined as the space available for functional use including a parent to stay seated, reclining or fully recumbent and excludes other defined spaces such as plumbing fixtures, anterooms, vestibules, toilet rooms, closets, lockers, wardrobes, fixed-based cabinets, and wall-hung counters

^Clear floor space is defined as the space available for functional use including a parent to stay seated, reclining or fully recumbent and excludes other defined spaces such as plumbing fixtures, anterooms, vestibules, toilet rooms, closets, lockers, wardrobes, fixed-based cabinets, and wall-hung counters

Appendix S10: AGREE-II

Author (Year)	Country	AGREE-II Domains and Evaluation						Overall quality	Overall assessment
		Scope and purpose	Stakeholder involvement	Rigour of development	Clarity and presentation	Applicability	Editorial independence		
Martin 1999(1)	USA	94%	56%	14%	100%	10%	4%	4	Not recommended[a]
Martin 2003(2)	USA	97%	69%	28%	100%	13%	8%	5	Not recommended[a]
Blanco Bravo 2004(3)	Spain	58%	19%	16%	67%	19%	54%	3	Not recommended[a]
White 2006(4)	USA	97%	69%	19%	100%	13%	8%	5	Not recommended[a]
White 2007(5)	USA	97%	81%	35%	100%	13%	8%	5	Not recommended[a]
Novoa 2009(6)	Chile	100%	64%	33%	100%	71%	100%	4	Not recommended[a]
White 2013(7)	USA	97%	69%	33%	100%	13%	71%	5	Not recommended[a]
Gracia 2013(9)	Spain	97%	17%	34%	86%	44%	100%	5	Recommended
White 2020(8)	USA	97%	69%	39%	100%	13%	17%	5	Not recommended[a]
Bajaña 2021(10)	Chile	100%	61%	31%	100%	65%	100%	4	Recommended
Altimier 2023(11)	USA	97%	56%	31%	100%	17%	92%	4	Recommended

Overall guideline quality assessment is rated from 1, lowest possible quality, to 7, highest possible quality, and a recommendation for or against use, or for with modifications.

References

1. Martin GI. Recommended Standards for Newborn ICU Design. Journal of Perinatology. 1999;19(S2):S2-S12. doi: 10.1038/sj.jp.7200233.
2. Martin GI. Recommended Standards for Newborn ICU Design. J Perinatol. 2003;23(S1):S1-S21.
3. Blanco Bravo D, Comite de Estandares de la Sociedad Espanola de Neonatologia, Junta Directiva de la Sociedad Espanola de Neonatologia. Niveles asistenciales y recomendaciones de minimos para la atencion neonatal [Levels of care and minimum recommendations for neonatal healthcare]. An Pediatr (Barc). 2004;60(1):56-64. doi: 10.1016/s1695-4033(04)78217-x. PubMed PMID: 14718132.
4. White RD. Recommended standards for newborn ICU design. Journal of Perinatology. 2006;26(S3):S2-S18. doi: 10.1038/sj.jp.7211587. PubMed PMID: 44489576.
5. White RD. Recommended standards for the newborn ICU. J Perinatol. 2007;27 Suppl 2(SUPPL. 2):S4-S19. doi: 10.1038/sj.jp.7211837. PubMed PMID: 18034180.
6. Novoa JM, Milad M, Vivanco G, Fabres J, Ramírez R. Recomendaciones de organización, características y funcionamiento en Servicios o Unidades de Neonatología. Revista chilena de pediatría. 2009;80(2):168-87.
7. White RD, Smith JA, Shepley MM, Committee to Establish Recommended Standards for Newborn ICUD. Recommended standards for newborn ICU design, eighth edition. J Perinatol. 2013;33 Suppl 1(SUPPL. 1):S2-16. doi: 10.1038/jp.2013.10. PubMed PMID: 23536026.
8. White RD, Consensus Committee on Recommended Design Standards for Advanced Neonatal C. Recommended standards for newborn ICU design, 9th edition. J Perinatol. 2020;40(Suppl 1):2-4. doi: 10.1038/s41372-020-0766-2. PubMed PMID: 32859957.
9. Rite Gracia S, Fernandez Lorenzo JR, Echaniz Urcelay I, Botet Mussons F, Herranz Carrillo G, Moreno Hernando J, et al. [Health care levels and minimum recommendations for neonatal care]. An Pediatr (Barc). 2013;79(1):51 e1- e11. Epub 20121221. doi: 10.1016/j.anpedi.2012.11.007. PubMed PMID: 23266243.
10. Galo Bajaña R, Carvajal EF, Cifuentes RJ, Gallardo CP, Kattan SJ, Mendez FA, et al. [Recommendations on organization, design, characteristics and operation of neonatology services]. Andes Pediatr. 2021;92(1):138-50. Epub 20210303. doi: 10.32641/andespediatr.v92i1.3553. PubMed PMID: 34106195.
11. Altimier L, Barton SA, Bender J, Browne J, Harris D, Jaeger CB, et al. Recommended standards for newborn ICU design. J Perinatol. 2023;43(Suppl 1):2-16. Epub 20231212. doi: 10.1038/s41372-023-01784-4. PubMed PMID: 38086961.
